# Translating Basic Research into Clinical Applications: Malaria Research at an NIH Lab

**DOI:** 10.1371/journal.ppat.1005190

**Published:** 2015-10-08

**Authors:** Xin-zhuan Su

**Affiliations:** Malaria Functional Genomics Section, Laboratory of Malaria and Vector Research, Division of Intramural Research, National Institute of Allergy and Infectious Diseases, National Institutes of Health, Bethesda, Maryland, United States of America

Malaria is a deadly disease caused by *Plasmodium* parasites with active transmission in nearly 100 countries. The parasites infect approximately 200 million people each year, killing over a half million patients. Although there is no active transmission in the United States, the US government and military have active research programs on malaria, including drug and vaccine development and various programs of basic research on parasite development, transmission, genetics, genomics, and disease virulence. Travelers and military personnel returning from malaria-endemic regions can be infected with the parasites, which may serve as the source of new transmission. In the US, there are many species of mosquitoes that can transmit the parasites. As one of the most advanced nations in science and technology in the world, we are in a good position (and have the responsibility) to develop disease-control measures and medicines to help cure and eventually eradicate the disease.

My laboratory at the National Institutes of Health (NIH) is interested in answering basic questions on malaria parasites and the diseases they cause, with a goal of translating the results from basic research into clinical applications. For example, drug resistance is a common problem for infectious diseases and cancer, and it is true of parasites as well. Chloroquine used to be a safe, effective, and affordable drug for treating malaria infections. However, drug-resistant parasites emerged after intensive use of the drug in the 1950s. A research project employing genetic techniques was launched at NIH, led by Dr. Thomas Wellems, to investigate the molecular mechanism of chloroquine resistance in the late 1980s. Mutations in a specific malaria gene were identified after approximately 15 years’ efforts. The identification of the mutations allows molecular surveillance of chloroquine resistance in endemic regions and helps disease-control organizations and doctors make appropriate decisions on drug use. The discovery of the gene has stimulated interest in studying the molecular mechanism of chloroquine resistance and provides experience and tools for investigating resistance to other drugs, including potential resistance to artemisinin. Artemisinin combination therapy (ACT) is the current treatment recommended by the World Health Organization (WHO) for the deadly *Plasmodium falciparum* malaria infection

In collaboration with scientists at the National Center for Advancing Translational Sciences (NCATS), my laboratory recently performed several large-scale screenings of chemical compound libraries to identify potential new medicines to treat malaria. These efforts have resulted in many potential compounds for blocking parasite transmission and for treating parasite infection. At the same time, we are using information generated from these studies to investigate parasite biology. We can treat the parasites with various compounds to disturb parasite metabolism and investigate their responses in growth and in gene expression. Analysis of parasite responses to a large number of compounds may lead to pathways the parasites employ to survive in the presence of specific compounds.

Another important research goal is to develop vaccines to prevent malaria infection. Unfortunately, we do not have an effective malaria vaccine at present, after more than 30 years of research and development. One of the difficulties is the lack of in-depth understanding of host–parasite interaction. As with human populations, each parasite isolated from a patient is likely to be unique, able to cause a disease different from, either slightly or dramatically, the one caused by another parasite. We are interested in studying the molecular basis of the diseases caused by malaria parasites. Considering the complexity and numerous factors in the field that can influence a disease outcome, including (but not limited to) variations in host and parasite genetic backgrounds, co-infections with viruses and/or bacteria, or nutritional status of the host, we have developed various rodent malaria models to dissect host–parasite interactions at the molecular level. In a recent study, we performed genetic crosses of a rodent malaria parasite called *Plasmodium yoelii*, infected mice with the same genetic background (inbred mice), and linked responses of many host genes to parasite genes or genetic loci. This work also led to the identification of many genes that play a role in host type I interferon response to infections. Currently we are working on dissecting the interactions of host and parasite genes. Results from these studies can later be tested in human infections in the field. Through these studies, we would like to identify the key pathways of host immune responses and the strategies the parasites use to evade host responses. We hope that these efforts will provide critical information for developing effective vaccines against malaria parasites in the future.

Scientific research is about studying something unknown; we cannot always expect a product in a specified time frame, as is done for assembling a TV set. Important discoveries often come from the ability to make a long-term commitment to major questions. However, all basic research efforts have the specific goal of translating the discoveries in a laboratory into products or cures for different diseases. Malaria research in my laboratory is no exception.

**Image 1:**
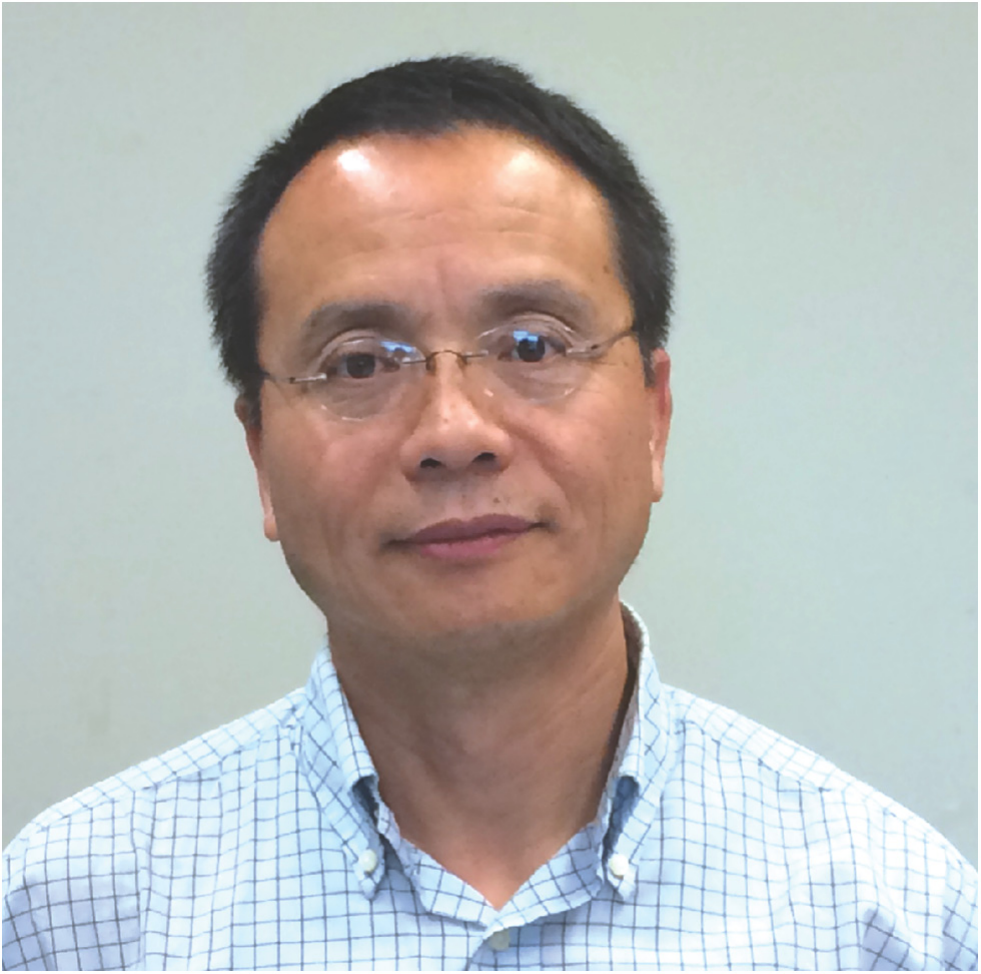
Xin-zhuan Su.

